# Disaggregating Asian-American Mortality in Drug-Related Overdoses and Behavioral Disorders: A Cross-Sectional Study

**DOI:** 10.1007/s40615-024-01983-5

**Published:** 2024-03-26

**Authors:** David T. Zhu, Anthony Zhong, Winnie J. Ho, Suzanne Tamang

**Affiliations:** 1 Medical Scientist Training Program, School of Medicine, Virginia Commonwealth University, 1201 E Marshall St, Richmond, VA 23298 USA; 2 Harvard Medical School, Boston, MA USA; 3 Yale School of Public Health, New Haven, CT USA; 4 Department of Medicine, Stanford University School of Medicine, Stanford, CA USA; 5 Department of Veterans Affairs, Program Evaluation Resource Center, Office of Mental Health and Suicide Prevention, Menlo Park, CA USA

**Keywords:** Asian American, Data disaggregation, Drug overdose, Mental disorders, Behavioral disorders, Disparities

## Abstract

Asian Americans have been historically underrepresented in the national drug overdose discourse due to their lower substance use and overdose rates compared to other racial/ethnic groups. However, aggregated analyses fail to capture the vast diversity among Asian-American subgroups, obscuring critical disparities. We conducted a cross-sectional study between 2018 and 2021 examining Asian-American individuals within the CDC WONDER database with drug overdoses as the underlying cause of death (*n* = 3195; ICD-10 codes X40–X44, X60–X64, X85, and Y10–Y14) or psychoactive substance–related mental and behavioral disorders as one of multiple causes of death (*n* = 15,513; ICD-10 codes F10–F19). Proportional mortality ratios were calculated, comparing disaggregated Asian-American subgroups to the reference group (Asian Americans as a single aggregate group). *Z*-tests identified significant differences between subgroups. Compared to the reference group (0.99%), drug overdose deaths were less prevalent among Japanese (0.46%; *p* < 0.001), Chinese (0.47%; *p* < 0.001), and Filipino (0.82%; *p* < 0.001) subgroups, contrasting with a higher prevalence among Asian Indian (1.20%; *p* < 0.001), Vietnamese (1.35%; *p* < 0.001), Korean (1.36%; *p* < 0.001), and other Asian (1.79%; *p* < 0.001) subgroups. Similarly, compared to the reference group (4.80%), deaths from mental and behavioral disorders were less prevalent among Chinese (3.18%; *p* < 0.001), Filipino (4.52%; *p* < 0.001), and Asian Indian (4.56%; *p* < 0.001) subgroups, while more prevalent among Korean (5.60%; *p* < 0.001), Vietnamese (5.64%; *p* < 0.001), Japanese (5.81%; *p* < 0.001), and other Asian (6.14%; *p* < 0.001) subgroups. Disaggregated data also revealed substantial geographical variations in these deaths obscured by aggregated analyses. Our findings revealed pronounced intra-racial disparities, underscoring the importance of data disaggregation to inform targeted clinical and public health interventions.

## Introduction

Asian Americans, Native Hawaiians, and Pacific Islanders (AANHPIs), comprising an estimated 20.6 million Asian Americans and 690,000 Native Hawaiians and Pacific Islanders, represents a diverse and rapidly growing population in the USA [1]. AANHPIs have been historically characterized as having the lowest rates of substance use and substance use disorders (SUDs) compared to other racial and ethnic groups in the USA, estimated at 4.6–4.7 individuals per 100,000 population in 2020 and 2021 [2]. However, such generalizations, dating back to the 1985 Heckler Report which found that “[t]he Asian/Pacific Island minority, in aggregate, is healthier than all racial/ethnic groups in the United States, including Whites,” fail to encapsulate the vast diversity of Asian communities and their lived experiences [3]. This is further reinforced by the “Model Minority Myth” regarding AANHPI communities, which may contribute to stigma experienced among AANHPIs when seeking comprehensive mental health and addiction treatment, and may potentially hamper progress to thorough investigations into the underlying factors perpetuating social and structural disparities in AANHPI drug overdoses [4]. Notably, stigma surrounding SUDs and help-seeking behaviors within AANHPI cultures may also exacerbate the data-sparse landscape of Asian-American SUD outcomes due to drug use underreporting and reluctance to participate in SUD research [5].

Aggregate data collection and reporting—relying on AANHPI data homogenization into a single monolithic group—have blurred the understanding of, and consequently hindered interventions aiming to address, critical health disparities among Asian American subgroups including in cardiovascular diseases, kidney diseases, and mental health needs, among others [6–8]. This can be partly attributed to small sample sizes of some Asian subpopulations, precluding more detailed analyses on inter-racial disparities [4]. However, there are also substantial health infrastructure challenges that lead to the underrepresentation on AANHPI populations in SUD research [9]. For instance, since 1997, federal agencies have adhered strictly to minimum reporting standards outlined by the Office of Management and Budget (OMB), resulting in the classification of AANHPI individuals into broad, aggregated categories. These rigid classifications overlook the intricate diversity within AANHPI subpopulations, hindering a nuanced understanding of substance use disorder (SUD) prevalence and patterns [9]. While aggregated data on SUDs among AANHPI individuals may highlight differences compared to other racial and ethnic groups, it fails to capture inter-racial disparities across subpopulations driven by social and structural factors such as socioeconomic status, cultural attitudes toward addiction and access to culturally competent addiction providers, stigma, access to detox/rehab programs, availability of healthcare resources in Asian languages, historical trauma, and more [4, 10, 11].

Recent estimates show that the burgeoning drug overdose crisis is claiming more than 100,000 lives annually in USA [12], representing an urgent public health concern. Amidst this rapidly growing and evolving public health epidemic, there lies a critical need to expand the discourse to incorporate Asian-American experiences and data on drug overdoses and mental or behavioral disorders within specific Asian-American subpopulations to investigate social and structural disparities. Without such systemic efforts, this undermines efforts toward achieving health equity and social justice and hinders broader initiatives aimed at addressing the root causes of substance use disorders within these communities. This study aims to fill a gap in the literature by examining deaths from drug-related overdoses and behavioral disorders among disaggregated Asian American subgroups on a national scale.

## Methods

This cross-sectional study analyzed 2018–2021 data from the Centers for Disease Control and Prevention Wide-Ranging Online Data for Epidemiologic Research (CDC WONDER) [13]. We described major trends in drug overdoses as the underlying cause of death, as defined by International Classification of Diseases, Tenth Revision (ICD-10) codes X40–X44, X60–X64, X85, and Y10–Y14, and psychoactive substance–related mental and behavioral disorders as one of multiple causes of death, as defined using ICD-10 codes F10–F19. Given the central focus of this study on generating preliminary insights into intra-racial health disparities among Asian-American subgroups, we did not further examine specific detailed causes of death related to psychoactive substance–related mental and behavioral disorders. Instead, our analysis of this variable remained broad as defined within CDC WONDER.

Overall and geographically stratified (by US census regions) proportional mortality ratios were calculated for various racial and ethnic groups in the USA (Hispanic and non-Hispanic White, Black, Asian, and American Indian and Alaskan Native (AIAN) individuals) to provide a broad overview of this issue in the USA. Notably, Asian-American individuals were included in our study as both a single aggregate group and disaggregated subgroups (Asian Indian, Chinese, Filipino, Japanese, Korean, Vietnamese, and other Asians).

To quantify observed differences in proportional mortality compared to the reference level (aggregated Asian Americans), we used a two-tailed *z*-test with a significance level of α = 0.05. All statistical analyses were performed using R (version 4.3.0). We adhered to STROBE reporting guidelines for cross-sectional studies. Finally, this study was exempt from Virginia Commonwealth University’s institutional review board, as it used CDC WONDER public use data and is compliant with data-use restrictions. The analyses were not pre-registered and the results should be considered exploratory.

## Results

Between 2018 and 2021, 322,907 deaths were documented among Asian-American individuals, with 3195 (0.99%) attributed to drug overdoses as the underlying cause and 15,513 (4.80%) linked to psychoactive substance–related mental and behavioral disorders as one of multiple causes **(**Tables 1 and 2**)**. We observed markedly higher proportions of deaths from drug overdoses among non-Hispanic White (2.47%; *p* < 0.001), non-Hispanic Black (3.40%; *p* < 0.001), AIAN (4.24%; *p* < 0.001), Hispanic (3.60%; *p* < 0.001), and multiracial (6.31%; *p* < 0.001) groups compared to the aggregated Asian-American reference group (0.99%) **(**Tables 1 and 2**)**. Similarly, we also observed increased proportional mortality from psychoactive substance–related mental and behavioral disorders among non-Hispanic White (15.02%; *p* < 0.001), non-Hispanic Black (11.79%; *p* < 0.001), AIAN (19.51%; *p* < 0.001), Hispanic (8.32%; *p* < 0.001), and multiracial (15.19%) groups compared to the aggregated Asian-American reference group (4.80%) **(**Tables 1 and 2**)**.

**Table 1:** Overall and sex-stratified proportional mortality from drug overdoses and mental or behavioral disorders (% all deaths within each racial and ethnic group), 2018–2021

Racial/ethnic group	Proportional mortality (*p*-value)			Proportional mortality ratio		
	Overall	Male	Female	Overall	Male	Female
Drug overdoses						
Non-Hispanic White	2.47% (*p* < 0.001)	3.11% (*p* < 0.001)	1.68% (*p* < 0.001)	2.49	2.24	3.15
Non-Hispanic Black	3.40% (*p* < 0.001)	4.45% (*p* < 0.001)	1.92% (*p* < 0.001)	3.43	3.21	3.59
Non-Hispanic AIAN	4.24% (*p* < 0.001)	4.67% (*p* < 0.001)	3.68% (*p* < 0.001)	4.28	3.36	6.87
Aggregate non-Hispanic AA (ref)	0.99%	1.39%	0.54%	1.00 (ref)	1.00 (ref)	1.00 (ref)
Non-Hispanic Asian Indian	1.20% (*p* < 0.001)	1.57% (*p* < 0.001)	0.61% (*p* = 0.210)	1.21	1.13	1.14
Non-Hispanic Chinese	0.47% (*p* < 0.001)	0.57% (*p* < 0.001)	0.37% (*p* < 0.001)	0.47	0.41	0.69
Non-Hispanic Filipino	0.82% (*p* < 0.001)	1.27% (*p* = 0.108)	0.38% (*p* < 0.001)	0.83	0.92	0.71
Non-Hispanic Japanese	0.46% (*p* < 0.001)	0.75% (*p* < 0.001)	0.26% (*p* < 0.001)	0.46	0.54	0.49
Non-Hispanic Korean	1.36% (*p* < 0.001)	1.77% (*p* < 0.001)	0.93% (*p* < 0.001)	1.37	1.28	1.74
Non-Hispanic Vietnamese	1.35% (*p* < 0.001)	1.82% (*p* < 0.001)	0.66% (*p* = 0.076)	1.36	1.32	1.22
Non-Hispanic Other Asian	1.78% (*p* < 0.001)	2.38% (*p* < 0.001)	0.93% (*p* < 0.001)	1.80	1.71	1.73
Non-Hispanic Multi-Racial	6.31% (*p* < 0.001)	7.30% (*p* < 0.001)	4.85% (*p* < 0.001)	6.36	5.26	9.06
Hispanic or Latino	3.60% (*p* < 0.001)	4.92% (*p* < 0.001)	1.80% (*p* < 0.001)	3.64	3.55	3.36
Mental or behavioral disorders						
Non-Hispanic White	15.02% (*p* < 0.001)	18.01% (*p* < 0.001)	11.84% (*p* < 0.001)	3.13	2.53	5.10
Non-Hispanic Black	11.79% (*p* < 0.001)	14.76% (*p* < 0.001)	8.48% (*p* < 0.001)	2.46	2.07	3.65
Non-Hispanic AIAN	19.51% (*p* < 0.001)	21.83% (*p* < 0.001)	16.75% (*p* < 0.001)	4.06	3.07	7.21
Aggregate non-Hispanic AA (ref)	4.80%	7.12%	2.32%	1.00 (ref)	1.00 (ref)	1.00 (ref)
Non-Hispanic Asian Indian	4.56% (*p* < 0.001)	6.73% (*p* = 0.021)	1.33% (*p* < 0.001)	0.95	0.94	0.57
Non-Hispanic Chinese	3.18% (*p* < 0.001)	5.09% (*p* < 0.001)	1.12% (*p* < 0.001)	0.66	0.72	0.48
Non-Hispanic Filipino	4.52% (*p* < 0.001)	6.94% (*p* = 0.261)	2.21% (*p* = 0.212)	0.94	0.97	0.95
Non-Hispanic Japanese	5.81% (*p* < 0.001)	8.58% (*p* < 0.001)	4.05% (*p* < 0.001)	1.21	1.21	1.75
Non-Hispanic Korean	5.60% (*p* < 0.001)	7.36% (*p* = 0.314)	4.09% (*p* < 0.001)	1.17	1.03	1.76
Non-Hispanic Vietnamese	5.64% (*p* < 0.001)	8.75% (*p* < 0.001)	1.29% (*p* < 0.001)	1.18	1.23	0.55
Non-Hispanic Other Asian	6.14% (*p* < 0.001)	8.61% (*p* < 0.001)	2.95% (*p* < 0.001)	1.28	1.21	1.27
Non-Hispanic Multi-Racial	15.19% (*p* < 0.001)	17.26% (*p* < 0.001)	12.65% (*p* < 0.001)	3.16	2.42	5.45
Hispanic or Latino	8.32% (*p* < 0.001)	10.97% (*p* < 0.001)	4.87% (*p* < 0.001)	1.73	1.54	2.09

*AIAN* American Indian or Alaskan Native, *AA* Asian American

**Table 2:** Age-stratified proportional mortality from drug overdoses and mental or behavioral disorders (% all deaths within each racial and ethnic group), 2018–2021

Racial/ethnic group	Proportional mortality (*p*-value)						Proportional mortality ratio					
	** ≤ **24 years	25–34 years	35–44 years	45–54 years	55–64 year**s**	≥ 65 years	≤ 24 years	25–34 years	35–44 years	45–54 years	55–64 years	≥ 65 years
Drug overdoses												
Non-Hispanic White	13.28% (*p* < 0.001)	35.86% (*p* < 0.001)	25.33% (*p* < 0.001)	10.53% (*p* < 0.001)	3.45% (*p* < 0.001)	0.19% (*p* < 0.001)	2.03	2.39	3.00	3.64	3.95	4.58
Non-Hispanic Black	4.30% (*p* < 0.001)	15.49% (*p* = 0.359)	13.21% (*p* < 0.001)	8.22% (*p* < 0.001)	4.24% (*p* < 0.001)	0.47% (*p* < 0.001)	0.66	1.03	1.56	2.84	4.86	11.52
Non-Hispanic AIAN	9.00% (*p* < 0.001)	17.14% (= *p* = 0.002)	13.09% (*p* < 0.001)	7.55% (*p* < 0.001)	3.08% (*p* < 0.001)	0.50% (*p* < 0.001)	1.38	1.14	1.55	2.61	3.53	12.40
Aggregate non-Hispanic AA (ref)	6.53%	15.02%	8.45%	2.89%	0.87%	0.04%	1.00 (ref)	1.00 (ref)	1.00 (ref)	1.00 (ref)	1.00 (ref)	1.00 (ref)
Non-Hispanic Asian Indian	6.00% (*p* = 0.445)	15.60% (*p* = 0.639)	7.50% (*p* = 0.215)	2.29% (*p* = 0.086)	0.50% (*p* = 0.007)	0.07% (*p* = 0.010)	0.92	1.04	0.89	0.79	0.57	1.84
Non-Hispanic Chinese	5.75% (*p* = 0.376)	11.01% (*p* = 0.0003)	4.68% (*p* < 0.001)	1.91% (*p* = 0. 003)	0.50% (*p* = 0.002)	0.07% (*p* = 0.003)	0.88	0.73	0.55	0.66	0.57	1.74
Non-Hispanic Filipino	6.28% (*p* = 0.835)	10.12% (*p* < 0.001)	6.87% (*p* = 0.032)	3.22% (*p* = 0.296)	1.22% (*p* = 0.005)	0.09% (*p* < 0.001)	0.96	0.67	0.81	1.11	1.40	2.10
Non-Hispanic Japanese	0.00% (*p* = 0.114)	22.58% (*p* = 0.061)	10.00% (*p* = 0.475)	4.76% (*p* = 0.005)	2.02% (*p* < 0.001)	0.10% (*p* < 0.001)	0.00	1.50	1.18	1.65	2.31	2.56
Non-Hispanic Korean	13.97% (*p* < 0.001)	25.33% (*p* < 0.001)	12.57% (*p* < 0.001)	3.91% (*p* = 0.028)	1.02% (*p* = 0.473)	0.13% (*p* < 0.001)	2.14	1.69	1.49	1.35	1.17	3.13
Non-Hispanic Vietnamese	7.61% (*p* = 0.306)	18.09% (*p* = 0.054)	12.46% (*p* < 0.001)	3.94% (*p* = 0.004)	0.75% (*p* = 0.458)	0.07% (*p* = 0.037)	1.16	1.20	1.48	1.36	0.86	1.79
Non-Hispanic Other Asian	6.02% (*p* = 0.432)	14.59% (*p* = 0.690)	9.22% (*p* = 0.238)	2.29% (*p* = 0.045)	0.79% (*p* = 0.545)	0.12% (*p* < 0.001)	0.92	0.97	1.09	0.79	0.91	2.85
Non-Hispanic Multi-Racial	8.85% (*p* < 0.001)	28.99% (*p* < 0.001)	20.53% (*p* < 0.001)	11.03% (*p* < 0.001)	4.25% (*p* < 0.001)	0.40% (*p* < 0.001)	1.36	1.93	2.43	3.82	4.87	9.76
Hispanic or Latino	8.77% (*p* < 0.001)	21.98% (*p* < 0.001)	15.08% (*p* < 0.001)	7.18% (*p* < 0.001)	2.97% (*p* < 0.001)	0.20% (*p* < 0.001)	1.34	1.40	1.79	2.48	3.39	4.89
Mental or behavioral disorders												
Non-Hispanic White	7.77% (*p* < 0.001)	26.26% (*p* < 0.001)	25.27% (*p* < 0.001)	23.80% (*p* < 0.001)	24.90% (*p* < 0.001)	12.50% (*p* < 0.001)	2.58	2.37	2.27	2.92	3.53	3.21
Non-Hispanic Black	2.73% (*p* < 0.001)	12.84% (*p* = 0.204)	15.66% (*p* < 0.001)	16.23% (*p* < 0.001)	17.69% (*p* < 0.001)	9.49% (*p* < 0.001)	0.91	1.16	1.41	1.99	2.51	2.44
Non-Hispanic AIAN	7.54% (*p* < 0.001)	22.84% (*p* < 0.001)	25.12% (*p* < 0.001)	24.80% (*p* < 0.001)	24.82% (*p* < 0.001)	15.83% (*p* < 0.001)	2.51	2.06	2.26	3.04	3.52	4.06
Aggregate non-Hispanic AA (ref)	3.01%	11.08%	11.11%	8.16%	7.05%	3.90%	1.00 (ref)	1.00 (ref)	1.00 (ref)	1.00 (ref)	1.00 (ref)	1.00 (ref)
Non-Hispanic Asian Indian	2.77% (*p* < 0.001)	11.58% (*p* = 0.663)	12.82% (*p* = 0.049)	9.85% (*p* = 0.003)	6.52% (*p* = 0.167)	3.20% (*p* < 0.001)	0.92	1.04	1.15	1.21	0.92	0.82
Non-Hispanic Chinese	2.63% (*p* < 0.001)	7.13% (*p* = 0.575)	6.41% (*p* < 0.001)	5.08% (*p* < 0.001)	5.27% (*p* < 0.001)	2.74% (*p* < 0.001)	0.88	0.64	0.58	0.62	0.75	0.70
Non-Hispanic Filipino	2.49% (*p* < 0.001)	7.56% (*p* = 0.465)	8.99% (*p* = 0.010)	6.91% (*p* = 0.010)	6.25% (*p* = 0.012)	3.88% (*p* = 0.862)	0.83	0.68	0.81	0.85	0.89	1.00
Non-Hispanic Japanese	0.00% (*p* < 0.001)	16.13% (*p* = 0.138)	10.00% (*p* = 0.672)	10.50% (*p* = 0.030)	10.80% (*p* < 0.001)	5.34% (*p* < 0.001)	0.00	1.46	0.90	1.29	1.53	1.37
Non-Hispanic Korean	4.93% (*p* < 0.001)	13.76% (*p* = 0.056)	13.89% (*p* = 0.024)	9.59% (*p* = 0.056)	7.84% (*p* = 0.125)	4.63% (*p* < 0.001)	1.64	1.24	1.25	1.18	1.11	1.19
Non-Hispanic Vietnamese	3.60% (*p* < 0.001)	9.72% (*p* = 0.445)	13.28% (*p* = 0.047)	10.04% (*p* = 0.001)	8.99% (*p* < 0.001)	4.13% (*p* = 0.075)	1.20	0.88	1.20	1.23	1.28	1.06
Non-Hispanic Other Asian	3.16% (*p* < 0.001)	13.73% (*p* = 0.784)	12.38% (*p* = 0.084)	8.29% (*p* = 0.799)	7.33% (*p* = 0.423)	4.73% (*p* < 0.001)	1.05	1.24	1.11	1.02	1.04	1.21
Non-Hispanic Multi-Racial	4.38% (*p* < 0.001)	16.51% (*p* < 0.001)	22.11% (*p* < 0.001)	20.62% (*p* < 0.001)	21.82% (*p* < 0.001)	12.90% (*p* < 0.001)	1.46	1.49	1.99	2.53	3.09	3.31
Hispanic or Latino	4.66% (*p* < 0.001)	16.72% (*p* < 0.001)	16.39% (*p* < 0.001)	12.80% (*p* < 0.001)	11.03% (*p* < 0.001)	5.80% (*p* < 0.001)	1.55	1.51	1.47	1.57	1.56	1.49

*AIAN* American Indian or Alaskan Native, *AA* Asian American

Disaggregating Asian Americans revealed significant intra-racial disparities between subgroups. Notably, Japanese (0.46%; *p* < 0.001), Chinese (0.47%; *p* < 0.001), and Filipino (0.82%; *p* < 0.001) subgroups exhibited lower proportions of fatal drug overdoses compared to the aggregated Asian reference group (0.99%). In contrast, Asian Indian (1.20%; *p* < 0.001), Vietnamese (1.35%; *p* < 0.001), Korean (1.36%; *p* < 0.001), and other Asian (1.79%; *p* < 0.001) populations exhibited higher proportions compared to the reference **(**Tables 1 and 2**)**.

Similarly, Chinese (3.18%; *p* < 0.001), Filipino (4.52%; *p* < 0.001), and Asian Indian (4.56%; *p* < 0.001) subgroups displayed lower proportions of deaths from psychoactive substance–related mental and behavioral disorders compared to the aggregated Asian reference group (4.80%). Conversely, Korean (5.60%; *p* < 0.001), Vietnamese (5.64%; *p* < 0.001), Japanese (5.81%; *p* < 0.001), and other Asian (6.14%; *p* < 0.001) populations exhibited higher proportional mortality compared to the reference **(**Tables 1 and 2**)**.

Furthermore, our analysis revealed significant regional disparities upon stratification by US census region. The aggregated Asian-American group demonstrated the highest proportions of fatal drug overdose in the Midwest (1.25%), followed by the South (1.11%), Northeast (1.09%), and West (0.87%); however, these geographical patterns varied considerably across Asian-American subgroups **(**Table 3**)**. Similarly, the aggregated Asian-American group displayed the highest proportions of deaths from psychoactive substance–related mental and behavioral disorders in the Midwest (7.22%), followed by the South (6.20%), Northeast (5.61%), and West (3.67%) **(**Table 3**)**. Yet, disaggregated data revealed that regionally stratified drug overdoses were most prevalent in the Northeast among Chinese, Vietnamese, and other Asian subgroups, while in the Western regions among Filipino and Japanese subgroups **(**Table 3**)**.

**Table 3:** US census region-stratified proportional mortality from d rug overdoses and mental or behavioral disorders ( % all deaths within each racial and ethnic group), 2018–2021

Racial/ethnic group	Proportional mortality ( *p*-value)					Proportional mortality ratio			
	Northeast	Midwest	South	West		Northeast	Midwest	South	West
Drug overdoses									
Non-Hispanic White	2.80% (*p* < 0.001)	2.19% (*p* < 0.001)	2.55% (*p* < 0.001)	2.34% (*p* < 0.001)	2.56		1.75	2.30	2.69
Non-Hispanic Black	4.40% (*p* < 0.001)	4.77% (*p* < 0.001)	2.59% (*p* < 0.001)	4.13% (*p* < 0.001)	4.04		3.82	2.33	4.75
Non-Hispanic AIAN	7.25% (*p* < 0.001)	5.44% (*p* < 0.001)	3.72% (*p* < 0.001)	3.94% (*p* < 0.001)	6.65		4.35	3.35	4.54
Aggregate non-Hispanic AA ( ref)	1.09%	1.25%	1.11%	0.87%	1.00 (ref)		1.00 (ref)	1.00 (ref)	1.00 (ref)
Non-Hispanic Asian Indian	1.25% (*p* = 0.122)	1.29% (*p* = 0.829)	0.95% (*p* = 0.094)	1.42% (*p* < 0.001)	1.15		1.03	0.86	1.63
Non-Hispanic Chinese	0.51% (*p* < 0.001)	0.63% (*p* = 0.001)	0.66% (*p* < 0.001)	0.40% (*p* < 0.001)	0.47		0.50	0.59	0.46
Non-Hispanic Filipino	0.74% (*p* = 0.019)	0.63% (*p* < 0.001)	0.79% (*p* = 0.011)	0.86% (*p* = 0.732)	0.68		0.50	0.71	0.99
Non-Hispanic Japanese	0.46% (*p* < 0.001)	0.89% (*p* < 0.001)	0.43% (*p* < 0.001)	0.44% (*p* < 0.001)	0.42		0.71	0.39	0.51
Non-Hispanic Korean	1.30% (*p* < 0.001)	2.15% (*p* < 0.001)	1.50% (*p* = 0.007)	1.18% (*p* < 0.001)	1.19		1.72	1.35	1.36
Non-Hispanic Vietnamese	1.90% (*p* < 0.001)	1.40% (*p* = 0.584)	1.37% (*p* = 0.020)	1.23% (*p* < 0.001)	1.74		1.12	1.23	1.41
Non-Hispanic Other Asian	2.26% (*p* < 0.001)	1.64% (*p* = 0.010)	1.64% (*p* < 0.001)	1.71% (*p* < 0.001)	2.07		1.31	1.48	1.97
Non-Hispanic Multi-Racial	8.64% (*p* < 0.001)	7.21% (*p* < 0.001)	5.66% (*p* < 0.001)	5.98% (*p* < 0.001)	7.93		5.77	5.10	6.87
Hispanic or Latino	6.17% (*p* < 0.001)	4.97% (*p* < 0.001)	2.26% (*p* < 0.001)	3.80% (*p* < 0.001)	5.66		3.98	2.04	4.37
Mental or behavioral disorders									
Non-Hispanic White	14.13% (*p* < 0.001)	17.30% (*p* < 0.001)	14.78% (*p* < 0.001)	13.19% (*p* < 0.001)		2.52	2.39	2.38	3.59
Non-Hispanic Black	12.06% (*p* < 0.001)	14.23% (*p* < 0.001)	11.40% (*p* < 0.001)	8.54% (*p* < 0.001)		2.15	1.97	1.84	2.33
Non-Hispanic AIAN	26.07% (*p* < 0.001)	28.42% (*p* < 0.001)	16.75% (*p* < 0.001)	17.61% (*p* < 0.001)		4.65	3.94	2.70	4.80
Aggregate non-Hispanic AA (ref)	5.61%	7.22%	6.20%	3.67%		1.00 (ref)	1.00 (ref)	1.00 (ref)	1.00 (ref)
Non-Hispanic Asian Indian	4.90% (*p* = 0.002)	5.73% (*p* < 0.001)	4.78% (*p* < 0.001)	3.03% (*p* < 0.001)		0.87	0.79	0.77	0.83
Non-Hispanic Chinese	4.53% (*p* < 0.001)	5.29% (*p* < 0.001)	4.93% (*p* = 0.049)	1.93% (*p* < 0.001)		0.81	0.73	0.79	0.53
Non-Hispanic Filipino	5.95% (*p* = 0.310)	5.27% (*p* < 0.001)	5.63% (*p* = 0.004)	4.08% (*p* < 0.001)		1.06	0.73	0.91	1.11
Non-Hispanic Japanese	7.42% (*p* = 0.006)	7.66% (*p* < 0.001)	7.51% (*p* < 0.001)	5.45% (*p* < 0.001)		1.33	1.06	1.21	1.49
Non-Hispanic Korean	6.21% (*p* = 0.072)	7.32% (*p* = 0.876)	8.23% (*p* < 0.001)	3.97% (*p* = 0.059)		1.11	1.01	1.33	1.08
Non-Hispanic Vietnamese	9.12% (*p* < 0.001)	10.72% (*p* < 0.001)	7.39% (*p* < 0.001)	3.17% (*p* = 0.001)		1.63	1.48	1.19	0.86
Non-Hispanic Other Asian	7.44% (*p* < 0.001)	9.53% (*p* < 0.001)	6.72% (*p* = 0.048)	4.05% (*p* = 0.007)		1.33	1.32	1.09	1.10
Non-Hispanic Multi-Racial	16.25% (*p* < 0.001)	19.91% (*p* < 0.001)	13.58% (*p* < 0.001)	14.37% (*p* < 0.001)		2.90	2.76	2.19	3.92
Hispanic or Latino	13.45% (*p* < 0.001)	16.83% (*p* < 0.001)	13.90% (*p* < 0.001)	11.52% (*p* < 0.001)		2.40	2.33	2.24	3.14

*AIAN* American Indian or Alaskan Native, *AA* Asian American

We conducted similar analyses on deaths among Native Hawaiian and Pacific Islander (NHPI) subgroups such as Hawaiians, Guamanians, Samoans, and other Pacific Islanders. However, given the substantial amount of data suppression for these NHPI subgroups within the CDC WONDER database, particularly when stratified across geographical regions, we did not include these as part of our findings.

## Discussion

Our cross-sectional analysis highlights substantial disparities in proportional mortality linked to drug overdoses and psychoactive substance–related mental and behavioral disorders across various racial and ethnic groups in the USA. Our findings further reinforce the idea that the use of a single monolithic group, “Asian and Pacific Islanders,” does not sufficiently describe the full extent of nuanced health outcomes and disparities across the over 17 million members of nearly 50 different races and ethnicities [14]. Consistent with prior research [2], a higher proportion of fatal drug overdoses and psychoactive substance–related mental and behavioral disorders was observed among non-Hispanic White, non-Hispanic Black, AIAN, Hispanic, and multiracial individuals compared to the aggregated Asian-American reference group. Moreover, our findings also shed light on significant intra-racial disparities across disaggregated Asian-American subgroups, a field of literature previously underrecognized due to relatively low overall rates of drug overdoses and racialized stereotypes that influence Asian-American health, including the model minority stereotype [4, 15].

Specifically, the lower proportions of deaths from drug overdoses and psychoactive substance–related mental and behavioral disorders observed among Chinese and Filipino subgroups suggest potential upstream social factors that may play a role in mitigating health risks within these communities [15]. In contrast, the higher proportions observed among certain subgroups, such as Koreans, Vietnamese, and other Asians, call for a deeper understanding of the unique challenges faced by these communities in relation to substance use and mental health. Our findings emphasize the importance of implementing enhanced data collection systems that capture racial and ethnic data with more granularity and nuance. To address such disparities, policymakers could prioritize the development of targeted intervention programs funded by agencies such as the Substance Abuse and Mental Health Services Administration (SAMHSA) and the Health Resources and Services Administration (HRSA) focus on culturally competent prevention and treatment strategies tailored locally to Asian-American communities at risk for drug overdose or mental and behavioral disorders. Such interventions should be tailored and informed by community-based participatory research and implemented based on the specific needs of these communities. Funding may be acquired from agencies such as the National Institute on Minority Health and Health Disparities (NIMHD).

By recognizing the heterogeneity within Asian-American demographics, our findings highlight the necessity of tailored interventions and policies that consider the unique cultural, socioeconomic, and regional factors influencing drug-related overdoses and behavioral disorders within these communities. We did not specifically examine these social and cultural factors and, thus, further research is warranted to better understand these factors that could increase the risk of drug overdoses and behavioral disorders within these communities. At the clinical level, healthcare providers should actively confront potential biases and microaggressions in treatment settings, alongside language barriers, to improve access to addiction treatments and mental health services among AANHPI populations for which such resources have been traditionally under-utilized [16, 17]. Furthermore, policymakers and community leaders may play pivotal roles in increasing access to culturally sensitive treatment services and dismantling systemic barriers to help-seeking; such efforts must be cognizant of the diverse ethnic backgrounds within AANHPI populations and tailor interventions to address SUD disparities accordingly [16, 17]. Furthermore, further efforts are needed by public health and epidemiological surveillance agencies to capture data on the upstream social and cultural factors that increase the risk of drug overdoses and behavioral disorders within AANHPI communities. Multisectoral partnerships involving governmental agencies such as the Centers for Disease Control and Prevention (CDC), the Department of Health and Human Services (HHS), and the Department of Housing and Urban Development (HUD) should be established to research and address upstream social determinants of health contributing to health risks within Asian-American communities.

Finally, the regional differences identified within the Asian-American population highlight the diverse geographical distribution of health risks. For instance, aggregate Asian-American deaths from psychoactive substance–related mental and behavioral disorders were concentrated in the Midwest and South, which notably differed from the disaggregated analyses which revealed that these deaths were most prevalent in the Northeast and West for the majority of Asian-American subgroups. These regional disparities may be explained by a multitude of factors—including increased illicitly manufactured fentanyl (IMF) penetration in the Northeast, variations in the availability and accessibility of substance use disorder treatment programs services and life-saving harm reduction resources such as naloxone, differences in law enforcement practices and policies surrounding substance use, and more—but further research is warranted [18, 19]. To improve data collection granularity and systems for understanding regional disparities, a multi-pronged approach is essential [6]. Firstly, federal agencies such as the CDC and OMH should collaborate to standardize data collection protocols, ensuring comprehensive reporting of racial and ethnic data with disaggregated categories for Asian-American populations. This may include implementing electronic health record systems that capture detailed demographic information and enhancing data-sharing capabilities among healthcare providers and public health agencies. In addition, partnerships with regional health systems and community-based organizations are essential to facilitate the collection of more granular data on social determinants that influence drug or substance use, enabling a more nuanced understanding of regional disparities [6]. Investing in health information technology infrastructure, supported by agencies such as the Office of the National Coordinator for Health Information Technology (ONC), may prove crucial for enabling interoperability and data exchange across systems. By strengthening data collection granularity and systems, policymakers, practitioners, and researchers can better tailor interventions and allocate resources to address regional disparities and inform programs aimed at preventing SUD-related morbidity and mortality within Asian-American subpopulations.

### Limitations

Several limitations should be recognized. First, the cross-sectional study design restricts our ability to establish causal relationships between observed disparities and factors contributing to drug-related overdose deaths and behavioral disorders within Asian-American communities. Second, our reliance on CDC WONDER data limited the scope of outcomes, covariates, and racial and ethnic categories, therefore, potentially overlooking crucial factors such as age, gender, socioeconomic status, access to health services, and the intersectionality of multiple Asian-American identities. Third, substantial amounts of data suppression for NHPI populations within the CDC WONDER database precluded our ability to accurately examine deaths from drug overdoses and mental or behavioral disorders; thus, future studies should aim to evaluate mortality trends across disaggregated NHPI subgroups using other regional and national databases. Finally, the absence of qualitative and patient-level data hinders a comprehensive understanding of contextual factors and individual experiences driving these disparities. Future research should address these constraints by utilizing more comprehensive data sources, longitudinal study designs, and a deeper exploration of social determinants and cultural contexts within Asian-American populations. For instance, future studies could be linked to social determinants of health data or more comprehensive public all-payer claims databases.

## Conclusion

Our findings highlight the complexity of understanding and addressing disparities in deaths from drug-related overdoses and behavioral disorders among Asian-American subgroups in the USA, emphasizing the necessity for disaggregating racial and ethnic data to inform targeted interventions. Future research employing more comprehensive data collection and granular analyses of Asian-American data in the context of drug overdoses and substance use disorders are warranted to promote health equity.

## Data Availability

Data collected and analyzed in this study are publicly available on the CDC WONDER website.
